# Specific correction of pyruvate kinase deficiency-causing point mutations by CRISPR/Cas9 and single-stranded oligodeoxynucleotides

**DOI:** 10.3389/fgeed.2023.1104666

**Published:** 2023-04-28

**Authors:** Sara Fañanas-Baquero, Matías Morín, Sergio Fernández, Isabel Ojeda-Perez, Mercedes Dessy-Rodriguez, Miruna Giurgiu, Juan A. Bueren, Miguel Angel Moreno-Pelayo, Jose Carlos Segovia, Oscar Quintana-Bustamante

**Affiliations:** ^1^ Division of Hematopoietic Innovative Therapies, Centro de Investigaciones Energéticas Medioambientales y Tecnológicas and Centro de Investigación Biomédica en Red de Enfermedades Raras (CIEMAT/CIBERER), Madrid, Spain; ^2^ Advanced Therapies Unit, Instituto de Investigación Sanitaria Fundación Jiménez Díaz (IIS-FJD, UAM), Madrid, Spain; ^3^ Servicio de Genética, Hospital Universitario Ramón y Cajal, IRYCIS and Centro de Investigaciones Biomédicas en Red de Enfermedades Raras (CIBERER), Madrid, Spain

**Keywords:** pyruvate kinase deficiency, point mutation, ssODN, CRISPR/Cas9, precise gene editing, allele specificity

## Abstract

Pyruvate kinase deficiency (PKD) is an autosomal recessive disorder caused by mutations in the *PKLR* gene. PKD-erythroid cells suffer from an energy imbalance caused by a reduction of erythroid pyruvate kinase (RPK) enzyme activity. PKD is associated with reticulocytosis, splenomegaly and iron overload, and may be life-threatening in severely affected patients. More than 300 disease-causing mutations have been identified as causing PKD. Most mutations are missense mutations, commonly present as compound heterozygous. Therefore, specific correction of these point mutations might be a promising therapy for the treatment of PKD patients. We have explored the potential of precise gene editing for the correction of different PKD-causing mutations, using a combination of single-stranded oligodeoxynucleotides (ssODN) with the CRISPR/Cas9 system. We have designed guide RNAs (gRNAs) and single-strand donor templates to target four different PKD-causing mutations in immortalized patient-derived lymphoblastic cell lines, and we have detected the precise correction in three of these mutations. The frequency of the precise gene editing is variable, while the presence of additional insertions/deletions (InDels) has also been detected. Significantly, we have identified high mutation-specificity for two of the PKD-causing mutations. Our results demonstrate the feasibility of a highly personalized gene-editing therapy to treat point mutations in cells derived from PKD patients.

## 1 Introduction

Pyruvate Kinase Deficiency (PKD) is a rare autosomal recessive non-spherocytic hemolytic anemia produced by mutations in the *PKLR* gene. This gene encodes for liver (LPK) and erythroid (RPK) pyruvate kinase proteins, which are expressed in the liver and in red blood cells (RBCs), respectively. RPK is particularly important in RBCs since it catalyzes the last step of the anaerobic glycolysis pathway, the main source of energy in these cells. The clinical signs of PKD range from mild to very severe anemia, reticulocytosis, splenomegaly, and iron overload. PKD can be life-threatening in severely affected patients (Grace et al., 2018). PKD prevalence is estimated at around 1:20,000, being higher in certain populations, such as the Amish community because of founder effect (Muir et al., 1984; Christensen et al., 2010). More than 300 mutations have been characterized up to now (Zanella et al., 2007; Bianchi and Fermo, 2020), seventy-two percent of those correspond to single substitutions occurring in the *PKLR* coding sequence (Grace et al., 2018; Bianchi and Fermo, 2020). Most variants are classified as missense and the rest as non-missense mutations. Although the pathological *PKLR* variants seem to alter domains critical for RPK structure and/or function, there is no clear relationship between PKD severity and a specific mutation. However, patients with two missense mutations might have a less severe prognosis (Grace et al., 2018). Some of the known variants have a high frequency, such as c.1436 G>A (p.Arg479His) mutation in Romani communities, c.1529 G>A (p.Arg510Gln) in 42% PKD patients in the United States, c.1456 C>T (p.Arg486Trp) in 41% European PKD patients and c.1468 C>T (p.Arg490Trp) mutation in 30% Asian PKD patients (Zanella et al., 2007; Grace et al., 2015). Importantly, PKD patients carry mutations in a compound heterozygous or homozygous state, however, carrier individuals bearing exclusively one *PKLR* pathological variant do not show clinical symptoms because of residual RPK activity (Van Wijk et al., 2009).

Current PKD management is palliative and includes transfusions, iron chelation, or splenectomy for severe cases. Recently, the allosteric activator Pyrukynd^®^ has been shown to be effective to ameliorate anemia in PKD patients with at least one missense mutation (Grace et al., 2019; Grace et al., 2019). Nevertheless, nowadays, the only curative option is allogeneic hematopoietic stem cell transplantation (HSCT). This therapy presents limitations in PKD patients due to the reduced availability of HLA-matched donors, infections, or high risk of severe graft-versus-host disease, which is especially relevant in PKD patients and leads to death in a significant percentage of patients (Henig and Zuckerman, 2014; Smetsers et al., 2016; van Straaten et al., 2017). Consequently, the autologous HSCT of genetically corrected cells would overcome all these limitations. With this in mind, lentiviral gene therapy for PKD has been demonstrated safe, with no adverse complications, and able to normalize hemoglobin levels in two PKD patients (NCT04105166) (López Lorenzo et al., 2020). Despite the promising results of the PKD lentiviral gene therapy, the ideal gene therapy approach should be based on the specific correction of the mutated gene. Gene editing allows the specific correction of the affected gene with a very limited genotoxic effect and entails the elimination of the mutated protein, which can interfere with the function of the therapeutic protein (Quintana-Bustamante et al., 2022). Gene editing has changed the therapeutical landscape of inherited hematopoietic diseases, from promising preclinical data to its success in clinical trials, mainly for hemoglobinopathies, such as β-thalassemia and sickle cell disease. In this therapeutic option, patient’s HSPCs are mobilized from their bone marrow niches to peripheral blood, purified, and *ex vivo* genetically modified, and then reinfused into the patient after being preconditioned to favor the engraftment of the gene edited cells (Quintana-Bustamante et al., 2022). *Ex vivo* gene editing of patient’s HSPCs has been shown promising results to correct red blood cell diseases (Canver et al., 2015; Brendel et al., 2016; Dever et al., 2016; Hoban et al., 2016; Ye et al., 2016; Lux et al., 2019; Fañanas-Baquero et al., 2021; Lattanzi et al., 2021; Newby et al., 2021; Wilkinson et al., 2021) and others inherited hematopoietic disorders (De Ravin et al., 2016, 2017; Pavel-Dinu et al., 2019; Román-Rodríguez et al., 2019; Schiroli et al., 2019; Ferrari et al., 2020; Rai et al., 2020) in preclinical research. Recently, gene editing is moving to the Clinics to correct hemoglobinopathies (Frangoul et al., 2021; Fu et al., 2022) (see ClinicalTrials.gov NCT03655678, NCT04208529, NCT04205435 and NCT04819841). For this reason, we have worked on the integration of a therapeutic cassette at the *PKLR* locus through homologous recombination mediated by nucleases to correct the energetic defect of PKD erythroid cells (Garate et al., 2015; Quintana-Bustamante et al., 2019; Fañanas-Baquero et al., 2021). However, the introduction of large homologous donor cassettes needed for therapeutic knock-in approaches is hampered in primitive HSPCs, unless p53 responses can be controlled (Schiroli et al., 2019; Ferrari et al., 2020; Sweeney et al., 2021) or suitable gene editing conditions for HSCs are used (Rai et al., 2023). Therefore, other approaches, such as the direct correction of PKD-causing mutations, should be explored. Currently, there are different gene editing strategies for targeting disease-causing mutations, such as the combination of the CRISPR/Cas9 system with single-stranded oligodeoxynucleotides (ssODNs). This system is simple to design and has shown promising results in correcting the sickle cell mutation in HSPCs (Hoban et al., 2015; DeWitt et al., 2016; Magis et al., 2019). On the other hand, the re-expression of fetal globins by gene editing is a true therapeutic alternative for the treatment of β-thalassemia and sickle cell disease (Canver et al., 2015; Brendel et al., 2016; Ye et al., 2016; Lux et al., 2019; Frangoul et al., 2021; Fu et al., 2022). More recently, base editors and prime editing starting to play an important role to correct specific mutations in HSPCs *ex vivo* (Newby et al., 2021; Liao et al., 2023) but also in vivo gene editing (Li et al., 2022).

In this work, we explore the potential of correcting PKD-causing mutations through the combination of mutation-specific guide RNAs (gRNAs) and ssODN to revert the targeted mutation and restore the wild-type amino acid sequence. We have assessed this system in two immortalized PKD patient-derived lymphoblastic cell lines (LCLs) and in hematopoietic stem and progenitor cells (HSPCs) carrying four different mutations. Our results showed precise correction in three of these mutations, with variable efficiency percentages and with the presence of additional insertions/deletions (InDels) that might be dependent on the activity of the CRISPR/Cas9 system. Moreover, we have identified a good level of correction for two of the PKD-causing mutations studied. Altogether, we demonstrate the feasibility of a highly personalized gene-editing therapy to treat PKD-causing mutations.

## 2 Materials and methods

### 2.1 Patient samples

All samples were collected under written consent from the donors and institutional review board agreement (SAF 2017-84248-P), and in accordance with the Helsinki Declaration of 1975, revised in 2000.

Mononuclear cells (MNCs) were purified using Ficoll-Paque PLUS (GE Healthcare) from PKD peripheral blood (PB) or bone marrow (BM) samples. The isolated MNCs were kept frozen in 10% dimethyl sulfoxide solution and stored in liquid nitrogen until being used. HSPCs were isolated according to their CD34^+^ expression using an Influx cell sorter (BD Biosciences). CD34^−^ cells were used for the generation of LCLs.

### 2.2 Generation of PKD lymphoblastic cell lines

Lymphoblastic cell lines from two different PKD heterozygous compound patients, carrying c.359 C >T/c.1168 G >A and c.1003 G >A/c.1456 C >T combinations of mutations respectively, were generated by transduction of CD34^−^ cells with Epstein-Barr virus solution in presence of cyclosporine A. The cultures were maintained in RPMI (Gibco/Thermo Fisher Scientific), 20% HyClone (GE Healthcare), 1% penicillin/streptomycin solution (Gibco), 0.005 mM β-mercaptoethanol (Gibco), 1 mM sodium pyruvate (Sigma-Aldrich) and non-essential amino acids (Lonza). Fresh media was added media every 10 days until the LCL cultures were established. LCLs were incubated under normoxic conditions: 37°C, 21% O_2_, 5% CO_2,_ and 95% relative humidity.

### 2.3 Design of gene editing tools

Mutation-specific gRNAs were designed using the design tool available from Integrated DNA Technologies’s website (https://eu.idtdna.com/site/order/designtool/index/CRISPR_CUSTOM), selecting the gRNAs with a high on-target score, low off-target score and the targeted mutation at their protospacer sequence. Different gRNAs were selected to target the different PKD mutations present in the PKD patient-derived cell (see Supplementary Table S1).

Single-stranded oligodeoxynucleotides (ssODN) donors were designed using SnapGene software (GSL Biotech LLC) according to the following criteria: 1) correct the targeted mutation by reverting to the wild-type amino acid sequence, 2) modifying several nucleotides to prevent the cutting of the gene-edited allele and facilitate the tracking of the gene-editing output, and 3) a symmetric structure of two homologous arms of 40 nucleotides approximately around the cutting site of its correspondent gRNA. Eleven ssODN donors were designed, some of them covered the same genomic sequence but in sense or antisense orientation (see Supplementary Table S1). Both gRNAs and ssODNs were acquired from Integrated DNA Technologies.

### 2.4 Nucleofection of gene editing tools

First, gRNA complexes were ensembled by combining Alt-R CRISPR-Cas9 crRNAs and Alt-R CRISPR-Cas9 tracRNA (both from Integrated DNA Technologies), heating at 95°C, and then, cooling at room temperature. Afterward, ribonucleoprotein (RNP) complexes were made by mixing gRNA complexes together with Alt-R HiFi Cas9 Nuclease (Integrated DNA Technologies). RNP complexes and ssODN were added into the nucleofection buffer and mixed with the cells prior to their nucleofection. PKD LCLs were nucleofected using EW-113 program with SF Cell Line 4D-Nucleofector X Kit (Lonza) in an Amaxa™ 4D-nucleofector (Lonza). After the electric pulse, the cells were incubated at 37°C and collected in pre-warmed media. Cells were maintained for 5 days, changing the media once every 2 days prior be analyzed. For HSPCs cells, P3 Primary Cell 4D-Nucleofector X Kit and EO-100 program was used.

### 2.5 Gene editing analysis

For clonal analysis of gene editing, genomic DNA from gene edited PKD LCLs was amplified by PCR with Herculase II Fusion DNA polymerase (Agilent Technologies), and the forward (CCC​ATA​CAG​TGC​CCA​TAC​AT) and reverse (AGA​TGT​GAG​TTC​TGA​GCC​CC) primers. The PCR amplicon was cloned using the Zero Blunt^™^ TOPO™ PCR Cloning Kit (ThermoFisher Scientific) and transformed into chemically competent TOP10 *E. coli*. Plasmid DNA of several bacteria colonies was purified, and Sanger sequenced.

For Next-Generation Sequencing (NGS) analyses, the genomic site of interest was amplified by PCR including Illumina adapters in the primer sequence using Herculase II Fusion DNA polymerase and the corresponding primers (Supplementary Table S2). NGS of the amplicons was performed by Genewiz with an estimated depth of 50,000 reads. Targeting efficacy was subsequently assessed by performing advanced analysis with Mosaic Finder software, a bioinformatic tool developed by the Genetics department at HRyC-IRYCIS that detects allelic mosaicism after NHEJ and HDR repair as previously described (Cervera et al., 2021; Alonso-Lerma et al., 2022; López-Márquez et al., 2022), and by Cas-Analyzer (Park et al., 2017).

For calculating gRNA activity, frequencies of InDels and the precise gene editing (HDR) were taken together. Additionally, for gRNA allele specificity, the proportion between the ratios of the wild-type allele frequency to the mutated allele frequency of the gene-edited condition and the control condition was calculated. When gRNA allele specificity is close to 1, there is no specificity of the gRNA for the mutated allele, but when it is greater than 1, the gRNA is specific for the mutated sequence.

### 2.6 *In vitro* erythroid differentiation

After gene-edited HSPCs, the cells were differentiated towards erythroid lineage using a 14-day protocol. Erythroid differentiation media was made by IMDM (Gibco), 2% HyClone (GE Healthcare), 0.5% P/S solution (Gibco-Thermo Fisher Scientific), 3% AB serum (Sigma-Aldrich), 10 μg/ml insulin (Sigma-Aldrich), 3U/ml heparin (Sigma-Aldrich), and 200 μg/ml holo-transferrin (Sigma-Aldrich). From day 0 to day 6, the cells were cultured in this basal media supplement with 3 U/ml EPO (Amgen), 10 ng/ml SCF (EuroBiosciences GmbH), 1 ng/ml human interleukin 3 (hIL-3) (Eurobiosciences), and 1 × 10^−6^ M dexamethasone (Sigma-Aldrich). On day 6, cell culture media was exchanged by the basal media with 3 U/ml EPO (Amgen) and 10 ng/ml SCF (Eurobiosciences) until day 9. In the last 5 days of the protocol, the media was supplemented with 3 U/ml EPO (Amgen) and an increased concentration of holo-transferrin (200 μg/ml). The cultures were maintained under normoxic conditions.

Immunophenotype of *in vitro* differentiated erythroid cells was analyzed using anti-human CD71-FITC and anti-human CD235a-PE in a BD LSRFortessa^™^ Cell Analyzer (BD Bioscience).

To quantify ATP production, CD235a^+^ erythroid cells were sorted and analyzed with CellTiter-Glo^®^ Luminescent Cell Viability Assay (Promega) according to the manufacturer’s instructions and the luminescence was recorded using Genius Pro reader (Tecan).

### 2.7 Statistical analysis

Statistical significance was determined using a two-tailed paired *t*-test when two variables were compared or ANOVA with Turkey’s multiple comparison test when more variables were compared with GraphPad Prism. The mean ± SD is shown in each graph. Additionally, the significance was represented by *p*-values: *p < 0.05, **p < 0.01, ***p < 0.001 and ****p < 0.0001.

## 3 Results

### 3.1 Correction of point mutations present in PKD patient-derived lymphoblastic cell line

**FIGURE 1:**
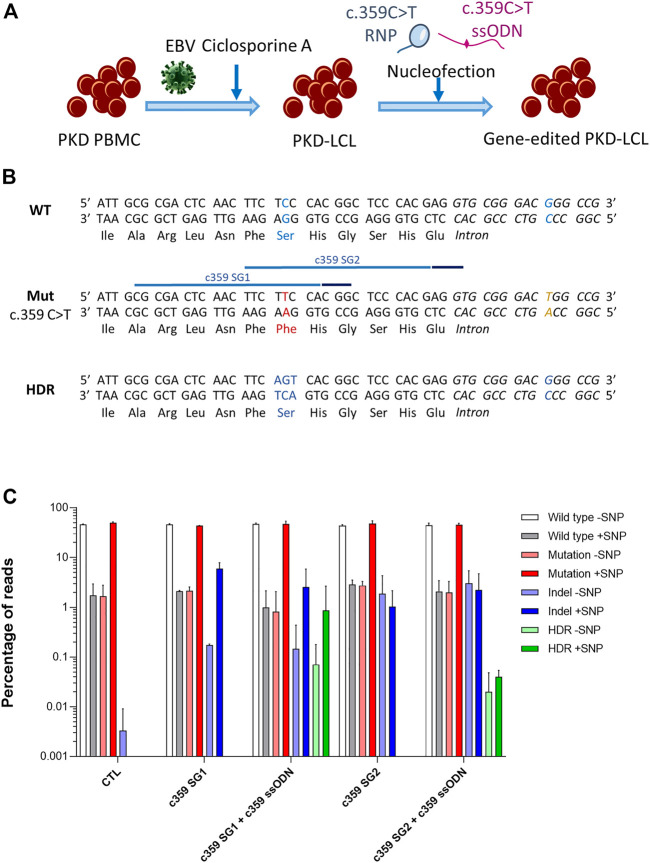
Correction of the c.359 C >T point mutations present in PKD-derived lymphoblastic cell line. **(A)** Scheme shows the generation of PKD lymphoblastic cell line and the nucleofection with the mutation-specific gene editing tools. **(B)** Genomic sequences of the wild-type allele show the wild-type nucleotide and amino acid in blue (top panel), the mutated allele with the c.359 C >T mutation and the mutated amino acid in red, along with the mutation-associated SNP in yellow (middle panel) with the target sequences of the two gRNA targeting the mutation (light blue line) and their PAM sequences (dark blue line), and the precise gene editing output with the modified nucleotides and reverted amino acid in blue (bottom panel). **(C)** Frequency of the different reads identified by Next-Generation Sequencing in PKD-LCLs gene edited with the specific gene editing tools for c.359 C >T mutation. Frequency values are represented as percentage of reads in the *y*-axis. Data are obtained from up to six independent experiments (N = 3 for CTL, N = 2 for c359 SG1, N = 9 for c359 SG1 + c359 ssODN and N = 2 for c359 SG2 and c359 SG2 + c359 ssODN). Data are presented by mean ± SD. Significance was analyzed by ANOVA with Turkey’s multiple comparison test.

We generated an immortalized lymphoblastoid cell line (LCL) derived from a PKD patient by infecting peripheral blood mononuclear cells (PB-MNCs) with Epstein Barr Virus (EBV) (Figure 1A). This PKD patient-cell line carried two heterozygous compound mutations, c.359 C >T and c.1168 G >A. As a proof-of-concept, we selected c.359 C >T mutation, which causes p. Ser120Phe conversion, to correct a PKD point mutation. We designed two guide RNAs (gRNAs, c359 SG1, and c359 SG2) targeting the mutated allele, and 80-mer single-stranded oligodeoxynucleotides (ssODN, c359 ssODN) to restore the wild-type amino acid sequence, introducing simultaneously a silent mutation to be able to track the desired modification (Supplementary Figure S1 and Supplementary Table S1). Interestingly, c.359 C >T mutation is three nucleotides away from c359 SG1 PAM sequence, but it is 15 nucleotides away from c359 SG2 PAM. Therefore, we expected c359 SG1 to be more mutation-specific than c359 SG2.

To assess the effectiveness of the designed tools to correct the c.359 C >T mutation, we nucleofected PKD LCLs with the corresponding RNP of each gRNA alone or together with the c359 ssODN. Five days later, we amplified the region around the mutation, cloned the PCR products into a pCR-Blunt II-Topo plasmid and transformed it into Top10 *E. coli* bacteria. We were able to analyze fourteen clones by Sanger sequencing (Supplementary Figure S1). Interestingly, g.5780G >T single nucleotide polymorphism (SNP) was identified at the mutated allele (6 out of 14 clones), 26 nucleotides downstream of the mutation. Furthermore, we identified three clones derived from PKD LCLs nucleofected with c359 SG1 + c359 ssODN, where the mutation had been replaced by the precise change introduced by the ssODN, and this modification appeared always with the SNP, confirming that the correction occurred in the mutated allele. However, no colony with precise modification was detected after c359 SG2 + c359 ssODN nucleofection. To quantify this in-depth, we performed Next-Generation Sequencing (NGS) (Figure 1C), and we found that most of the reads corresponded to either wild-type sequences without the SNP (WT-SNP) or the mutation carrying the SNP (Mut + SNP) in the control PKD LCLs, but there was a small representation of recombinant reads (WT + SNP and Mut-SNP). As expected, most of the InDels generated by c359 SG1 were identified in the same allele where the SNP was present (5.95 ± 1.93% reads had InDels and SNP). On the contrary, a small percentage of InDels were detected in the other allele without SNP (0.18 ± 0.01% reads), indicating that gRNA targeted the mutated sequence preferentially. In contrast, c359 SG2 showed less activity and specificity for the mutated sequence, with only 1.03 ± 1.14% reads with InDels together with the SNP and 1.88 ± 2.45% reads with InDels without the SNP. On the other hand, when we looked for the presence of the precise gene-edited sequence (HDR) in samples nucleofected with the c359 SG2 + c359 ssODN, we could only find it below the detection limit of our NGS analysis (0.07 ± 0.02% of the reads), with a little allele specificity (0.02 ± 0.84% HDR with SNP vs 0.05% HDR without SNP), these values are below the detection limit of our NGS analysis. On the other hand, more than 0.9% of the reads present in samples edited with the c359 SG1 + c359 ssODN carried the precise gene-edited sequence, with most of the edited alleles carrying the SNP (0.86 ± 1.81%) and very few edited alleles without the SNP (0.07 ± 0.11%). These data indicated that the combination of c359 SG1 and c359 ssODN can specifically edit the PKD-causing c.359 C>T mutation to revert it towards the wild-type amino acid sequence.

### 3.2 Design of gene editing tools to correct different PKD point mutations

To assess the feasibility of correcting different PKD-causing point mutations, we designed mutation-specific gRNAs together with ssODNs and generated an additional LCL derived from a different PKD patient (PKD LCL 2) that carries two different heterozygous compound mutations, c.1003 G >A and c.1456 C >T. These two mutations cause a p. Val335Met and p. Arg486Trp. We designed two new gRNAs and ssODNs targeting each of these two mutations and the c.1168 G >A mutation (p.Asp390Asn), which is also present in the previous case (PKD LCL 1) (see Supplementary Table S1 and Supplementary Figure S2). Due to the long distance between the cutting sites of each pair of gRNAs, a specific ssODN was designed for each gRNA, with the intention of keeping the cutting sites in the central area of the region covered by the ssODN. Additionally, sense (ssODN+) and antisense (ssODN-) ssODNs were generated to evaluate whether the ssODN orientation had a role in HDR frequency.

**FIGURE 2:**
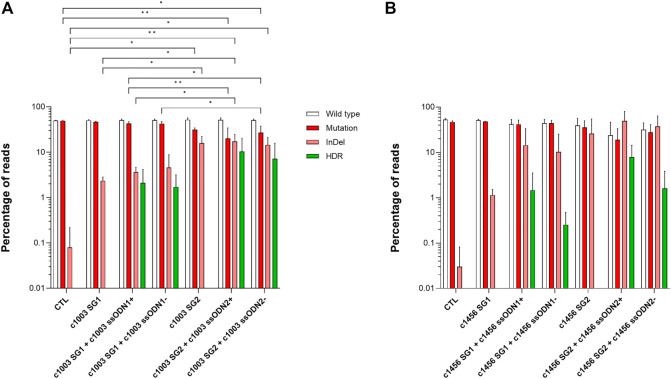
Correction of different point mutations present in a second PKD patient **(A)** Frequency of the different reads identified by Next-Generation Sequencing in PKD-LCLs gene-edited with the specific gene editing tools for c.1003 G >A mutation **(B)** Frequency of the different reads identified by Next-Generation Sequencing in PKD-LCLs gene-edited with the specific gene editing tools for c.1456 C>T mutation. Data are obtained from three independent experiments. Data are presented by mean ± SD. Significance was analyzed by ANOVA with Turkey’s multiple comparison test.

We nucleofected PKD LCL 1 with each combination of gRNA + ssODN targeting the c.1168 G >A mutation. Surprisingly we were not able to detect any reads with HDR using NGS (Supplementary Figure S2). However, up to 10% HDR was detected when the PKD LCL 2 was nucleofected with the gene editing tools that target c.1003 and c.1456 mutations (Figure 2A, B). Considering that the most active gRNAs were c1003 SG2 (16.0 ± 6.3%) and c1456 SG2 (26.1 ± 28.6%), we found out that the frequency of HDR mediated by these gRNAs was also the highest with 10.3 ± 10.1% for c1003 SG2 + c1003 ssODN2+, and 7.9 ± 6.5% for c1456 SG2 + c1456 ssODN2+. Interestingly, there was a trend for a higher HDR rate when sense ssODNs were used. The HDR ratio in the oligo sense *versus* antisense condition for the c1003 SG1 was 1.2 (HDR frequency c1003 SG1 + c1003 ssODN1+ vs. c1003 SG1+c1003 ssODN1-); for the c1003 SG2 was 1.4, for the c1456 SG1 was 5, and for the c1456 SG2 it was 4.9. Altogether, these results show that we were able to design different combinations of gRNAs and ssODNs to correct PKD-causing mutation accurately.

### 3.3 Mutation specificity to correct PKD point mutations

**FIGURE 3:**
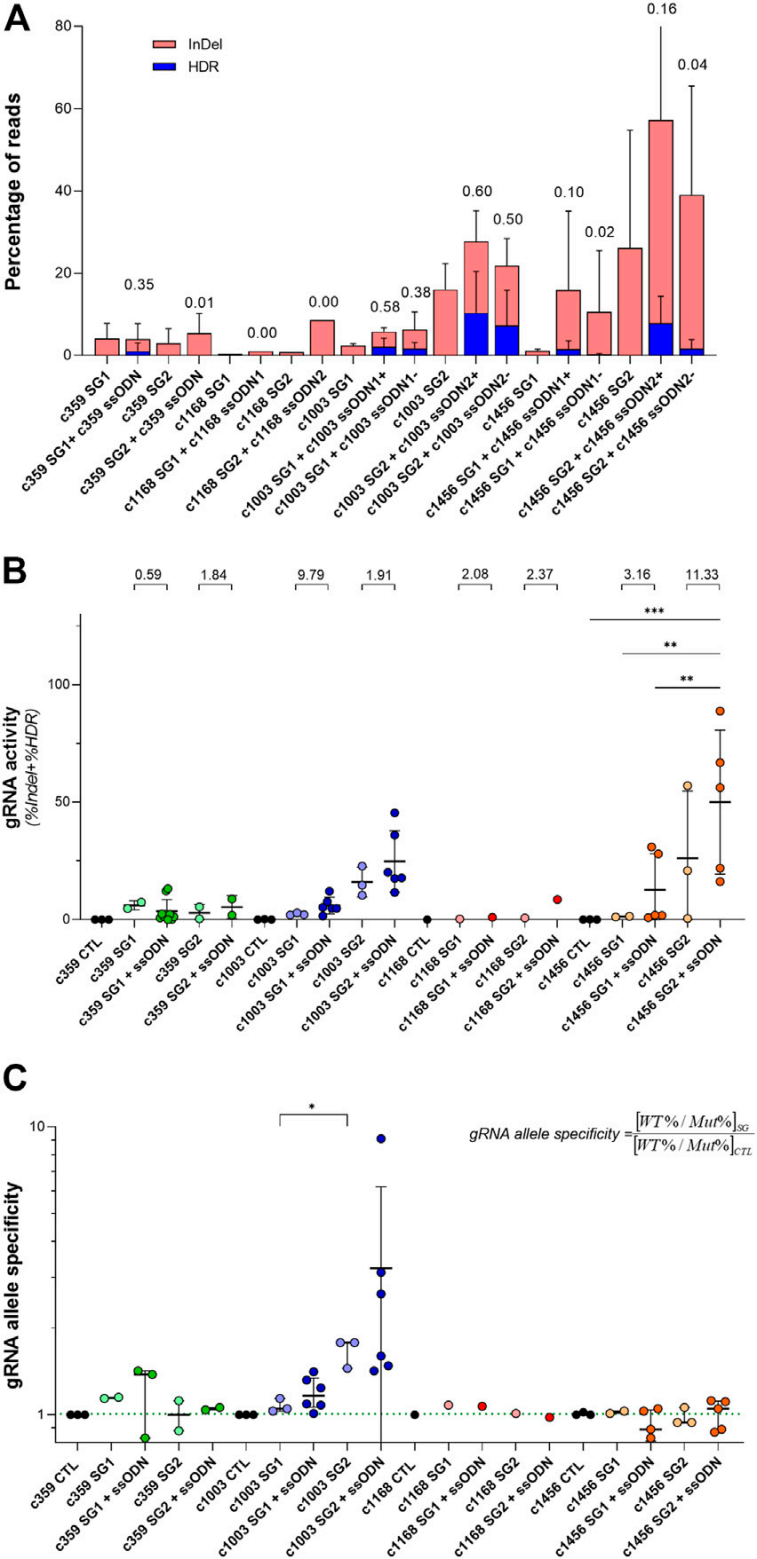
Analysis of the activity and mutation-specificity of the gene editing tools to correct PKD-causing mutation **(A)** Percentage of the gene-editing outputs, InDel in red and precise modification (HDR) in blue of the specific gene editing tools for c.359 C >T, c.1168 G >A, c.1003 G >A, and 1456 C >T. The ratio of HDR to the total of the gene-edited reads (InDel + HDR) is shown at the top of the bars **(B)** gRNA activity for the specific gRNAs for c.359 C >T, c.1168 G >A, c.1003 G >A, and c.1456 C >T. gRNA activity is defined as the total of the gene-edited reads (InDel + HDR). The ratio of gRNA activity with ssODN to the gRNA activity without ssODN for each condition is indicated at the top of the graph **(C)** gRNA allele specificity for c.359 C >T, c.1168 G >A, c.1003 G>A, and c.1456 C >T. gRNA allele specificity is defined as the ratio between the frequency of wild-type and mutated alleles in cells treated with the gRNA and the control. This value will be close to one when there is no specificity of a gRNA for the mutated allele and greater than one when it is specific for the mutated sequence. The dotted line indicates the gRNA allele specificity valor of 1. Data are obtained from the independent experiments shown in Figures 1, 2. Data are presented by mean ± SD. Significance was analyzed by ANOVA with Turkey’s multiple comparison test. The significance was represented by *p*-values: *p < 0.05 and **p < 0.01.

Looking at the previous data, we observed that the relationship between the frequency of InDels and the HDR depends considerably on the gRNA. As shown in Figure 3A, there were some conditions where the HDR frequency was similar to the InDel frequency, as occurred with the c359 SG1 (3.5 reads with HDR per every 10 reads with InDels), the c1003 SG1 or the c1003 SG2. However, the contribution of HDR in other conditions was minimal compared to the total gene editing outcomes, as happened with c359 SG2 (1 specie with HDR per every 100 reads with InDels), c1168 SG1 or c1168 SG2. In addition, c1456 SG1 or c1456 SG2 showed an intermediate HDR vs. InDels ratio. In addition, we could group the gRNAs similarly according to the distance of their PAM sequence to the mutation (Figure 1B and Supplementary Figure S2). c359 mutation was four nucleotides away from the c359 SG1 PAM, c1003 mutation was separated seven nucleotides from c1003 SG1 PAM, and one from c1003 SG2 PAM. On the contrary, the c359 mutation was fifteen nucleotides away from the c359 SG2 PAM, and the c1168 mutation was nineteen nucleotides apart from either the c1168 SG1 PAM or the c1168 SG2 PAM. And the c1456 mutation was eleven nucleotides separated from the c1456 SG1 PAM, and thirteen nucleotides from the c1456 SG2 PAM. Therefore, we can conclude that the ratio between the frequency of HDR and of InDels depends on the distance of the mutation to the PAM sequence of each of the gRNAs.

Interestingly, when the total activity of each of the gRNAs as the sum of InDels and HDR frequencies was considered (Figure 3B), we observed that regardless of the gene editing efficacy of each gRNA, the addition of ssODN increased its activity with respect to the same gRNA without ssODN. On the other hand, we were able to observe that certain gRNAs presented a higher editing activity for the mutated allele than for the wild-type allele. To quantify it, we defined the variable gRNA allele specificity (Figure 3C) as the ratio between the frequency of wild-type and mutated alleles in cells treated with the gRNA and the control. This value will be close to one when there is no specificity of a gRNA for the mutated allele and greater than one when it is specific for the mutated sequence. The gRNA allele specificity for the c359 SG1 and the c1003 SG2, whose PAM sequences were very close to the mutation, were higher than 1, 1.14 ± 0.01 and 1.67 ± 0.19 respectively. For the rest of the gRNAs, the PAMs were more than ten nucleotides away from the mutations and consequently, the gRNA allele specificity values were 1, showing no preference for the editing of the mutated allele over the wild-type allele. Our data suggest that in order to increase mutation specificity and promote HDR, the mutation must be very close to the gRNA PAM sequence.

### 3.4 Gene editing in hematopoietic stem and progenitor cells

**FIGURE 4:**
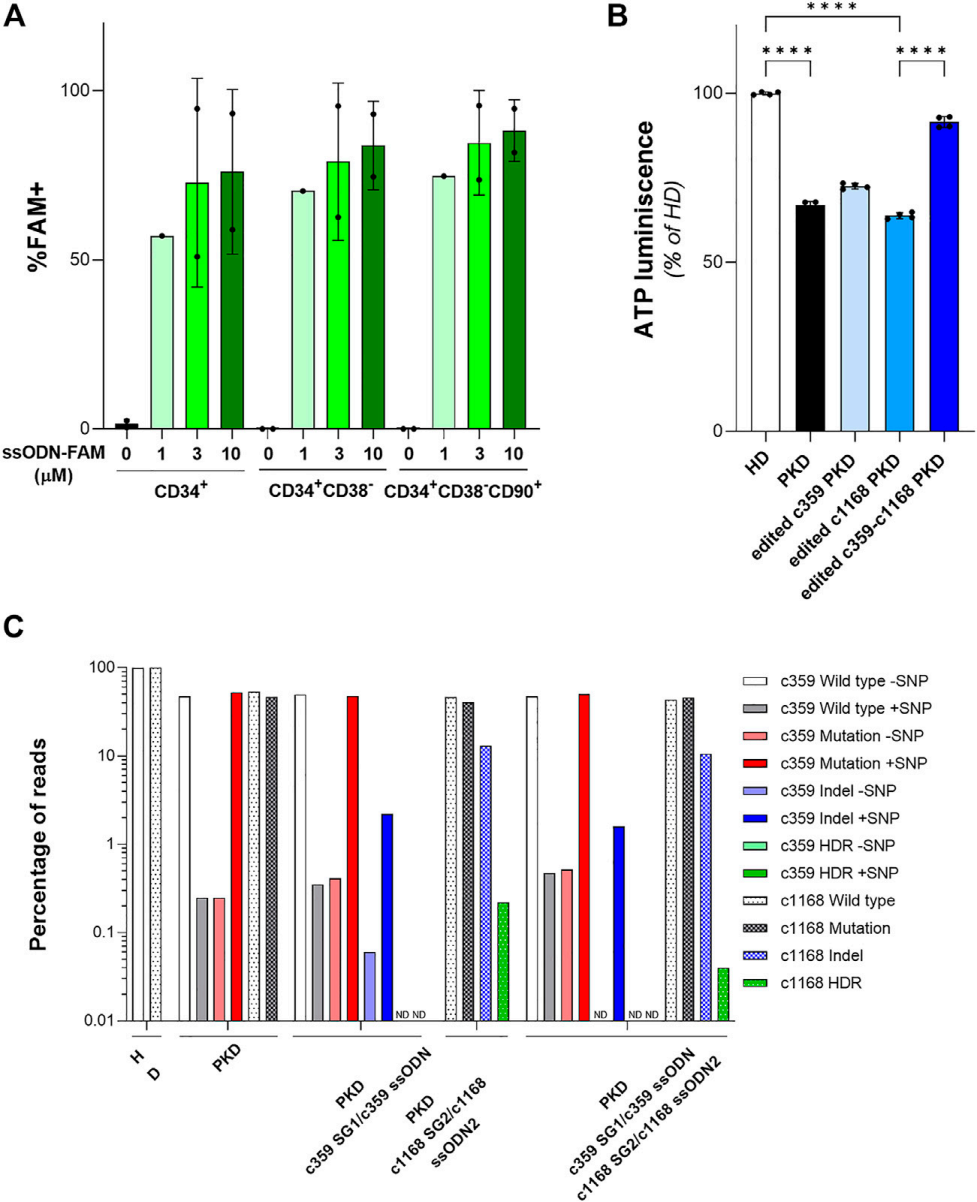
Gene editing in hematopoietic stem and progenitor cells (HSPCs) with gRNA and ssODN targeting PKD-causing mutations. **(A)** Percentage of FAM^+^ HSPCs after being nucleofected with different concentrations of ssODN-FAM. The immunophenotype of the HSPCs was analyzed. Data are obtained from 2 independent experiments. **(B)** ATP quantification of erythroid cells derived from PKD-HSPCs of a single patient differentiated *in vitro* after gene editing. Data are obtained from 3 technical replicates. **(C)** Frequency of the different reads identified by Next Generation Sequencing in erythroid cells derived from PKD-HSPCs of a single patient differentiated in vitro after gene editing with the specific gene editing tools for c.359 C>T and c.1168 G>A mutations. Data are presented by mean ± SD. Significance was analyzed by ANOVA with Turkey’s multiple comparison test. The significance was represented by *p*-values: ****p < 0.0001.

Once, we were able to design specific gene editing tools to revert PKD-causing mutations in PKD LCLs, we assessed their efficacy to correct HSPCs. To test the ability of the ssODN to be delivered in HSPCs, we nucleofected cord-blood CD34^+^ cells after 48 h pre-stimulation, with 1µM, 3 µM or 10 µM of a fluorescent oligonucleotide (ssODN-FAM). The frequency of FAM^+^ cells within the HSPC subsets was assessed by flow cytometry (Figure 4A and Supplementary Figure S3). We did not detect any increment in cell death associated with the highest concentration of ssODN-FAM. Additionally, the percentage of cells positive for FAM was similar at three or 10 µM. Furthermore, the frequency of FAM^+^ cells within the most primitive CD34^+^CD38^−^CD90^+^ population was 84.65 ± 15.49% at 3 µM, pointing out that ssODN is a suitable platform to perform gene editing on the most primitive population of HSPCs.

To confirm the feasibility of this gene editing approach to correct PKD-causing mutations, we purified CD34^+^ cells from splenic MNCs of the PKD patient that carries the two heterozygous compound mutations c.359 C >T and c.1168 G >A. After 2 days of pre-stimulation, PKD HSPCs were nucleofected with c359 SG1+3 µM c359 ssODN, c1168 SG2 +3 µM c1168 ssODN2, or both combinations of gRNA and ssODN together, to correct both mutations simultaneously (Supplementary Figure S3). Both sgRNAs were the most efficient to gene editing, either to promote HDR or to induce InDels, these two mutations present in PKD LCL1 cells. Nucleofected PKD HSPCs were *in vitro* differentiated into erythroid cells for 2 weeks. All conditions efficiently differentiated towards the erythroid lineage and expressed the CD71 and CD235a markers (Supplementary Figure S3). We sorted the CD235a^+^ population and assessed the ATP of those cells by luminescence assay. As expected, erythroid cells derived from PKD CD34^+^ cells showed a reduced ATP content in comparison with erythroid cells derived from healthy CD34^+^ cells (Figure 4B). Additionally, cells edited with each mutation-specific gene editing set had slightly higher ATP content than unmodified PKD cells. Furthermore, cells edited for both mutations showed similar ATP content as compared to cells derived from HSPCs from healthy donors. Surprisingly, when the CD235a^+^ population was analyzed by NGS, we detected very low HDR frequency in the differentiated population (Figure 4C). HDR at the c.359 mutation was not detected and the level of HDR at the c.1168 mutation was 0.22% or 0.04% when gene editing tools to correct only the c.1168 mutation and both mutations together respectively were used. Additionally, the frequency of InDels generated by the c359 SG1 was around 2% and up to 13% by the c1168 SG2. The high InDel frequency detected at c1168 G >A mutation in erythroid cells derived from PKD CD34^+^ cells after gene editing both mutations simultaneously might ameliorate the potential role of this mutation to block the RPK tetramer function (Valentini et al., 2002). On the other hand, the c359 SG1 maintained its mutation specificity in HSPCs, since the mutated allele was targeted preferentially (see Figure 4C). These data indicate the gRNA + ssODN combinations developed in PKD-LCLs were able to moderate edit PKD-causing mutations in HSPCs from PKD patients.

## 4 Discussion

We have designed different combinations of gRNAs and ssODN to correct four PKD-causing mutations. The designed gene editing tools were tested in patient-derived LCLs to assess their activity to target and correct specifically the PKD-causing mutations. The activity of the gRNAs depended on the target sequence. However, its mutation specificity and ability to promote HDR relied on the distance between the mutation and the PAM sequence. Patient-derived LCLs were a good platform to select the most effective combination of gRNAs and ssODNs to target PKD-causing mutation in primary HSPCs, revealing the relevance of our approach to develop clinically relevant gene editing tools to correct PKD-causing mutations.

The combination of gRNAs targeting PKD-causing mutations with ssODNs capable of reverting the mutated amino acid sequence, we were able to edit up to 10% of alleles with the c.1003 mutation (c1003 SG2 + c1003 ssODN2 + or c1003 ssODN2-) or the c.1456 mutation (c1456 SG2 + ssODN2+) (Figure 2). Additionally, two of the tested gRNAs, such as the c359 SG1 or the c1003 SG2 gRNAs, showed a very high mutation specificity, with a ratio of 1.40 ± 0.03 and 3.23 ± 2.96 allele specificity, respectively (Figure 3C). Although HDR events capable of correcting PKD mutation were detected, this precise correction was accompanied by an important frequency of InDels. Above 50% of the gene-edited alleles carried InDels at the gRNA target sequence (Figure 3A). Furthermore, when these gene editing tools were delivered to human HSPCs (Figure 4A), they could revert PKD-causing mutations in PKD CD34^+^ cells, although further improvements are needed to increase their efficiency of the process.

Up to now, more than 300 different variants in the *PKLR* gene have been described as the cause of PKD, most of which are found in the coding sequence region distributed throughout the gene (Zanella et al., 2007; Bianchi and Fermo, 2020). Most PKD patients are compound heterozygous for two mutations. Although the pathological *PKLR* variants seem to alter domains critical for RPK structure or function, there is no clear relationship between PKD severity and the occurrence of a specific mutation, however, patients with two missense mutations trend to have a less severe prognosis (Grace et al., 2018). We developed gene editing tools to modify four different missense mutations, all of them classified as pathogenic and recessive (https://databases.lovd.nl/shared/variants/PKLR/unique), present in two PKD patients. These mutations show a high frequency in PKD patients, since the c.1168 G >A has been reported in two out of 74 PKD cases and the c.1456 C >T in 20 out of 2,795 cases and four out of 616 healthy donors. Therefore, our gene editing approach could benefit multiple PKD patients. Additionally, the correction of any of these mutated alleles implies the correction of the PKD phenotype in the patient’s cells. Therefore, we can envision a set of different mutation-specific sets of gRNAs and ssODNs, which alone or in combination might be used to treat compound heterozygous PKD patients. On the other hand, mutation-specific gRNAs will be able to gene edit the mutation without altering the wild type allele, which might cause new disease-causing mutations in the gene edited wild type allele. This point becomes more relevant when patients carrying heterozygous mutations must be treated.

Based on our data, we can conclude that the efficiency and specificity of the editing of a mutation depends on its distance from the PAM sequence of the gRNA as well as on its activity (Figure 3). Consequently, very active gRNAs specifically targeting the mutated sequence with their PAM close to the mutation should be selected to generate mutation-specific gene editing tools. However, this point might be a constraint, since gRNA activity is highly dependent on its target DNA sequence, and highly active gRNAs directed against the mutation might not be selectable. Additionally, we did not find a preference in ssODN orientation to enhance HDR, since sense or antisense ssODNs for the c1168 and the c1456 mutations got similar HDR frequencies (Figure 2 and Supplementary Figure S2). Moreover, gRNA overall activity (Figure 3) was improved after adding ssODN into the nucleofection solution, in consistency with previous studies (Shapiro et al., 2020). The reason is unclear, but we hypothesize that an increment in the negative charge of the RNP complex might facilitate the action of the CRISPR/Cas9 system.

As shown in Figure 1 and Supplementary Figure S1, HDR mediated by ssODN in our system occurred locally. The c359 ssODN was able to revert the c359 C >T mutation, but g.5780 G >T SNP was kept unmodified after gene editing. In this way, we speculate that smaller ssODNs are enough to correct point mutations. Interestingly, this is consistent with the single-strand template repair (SSTR) mechanism (Gallagher et al., 2020; Ferenczi et al., 2021), where only a few bases around the DSB are modified. If this point is confirmed in future studies, it would open the possibility of increasing the frequency of specific correction of mutations by manipulating this repair pathway.

As previously mentioned, the frequency of InDels is important and accounts for more than half of the edited alleles (Figure 3A). Consequently, the precise correction might be enhanced by reducing the non-homologous end-joining pathway (NHEJ), which seems to compete with the precise correction as it is shown in Figure 3A. Different approaches to correct specific mutations have been developed, such as base editing (Komor et al., 2016; Gaudelli et al., 2017; Li et al., 2021; Newby and Liu, 2021; Li et al., 2022; Liao et al., 2023) or prime editing (Anzalone et al., 2019), which should be explored to correct PKD in the future. These other gene editing approaches could benefit from mutation-specific gRNAs to correct the altered allele specifically. On the other hand, we might see NHEJ as an opportunity to knock out the uncorrected alleles, consequently, the therapeutic effect of the precise correction might be improved by the NHEJ repair. This effect has been previously reported in gene-edited cells of congenital muscular dystrophies patients bearing the p. Gly293Arg *COL6A1* variant where the correction of the mutant allele by homologous-directed repair (HDR) occurred at a very low frequency (1%); however, the presence of frameshift variants and others resulting of NHEJ provoked the specific silencing of the mutant allele (40% of reads), with no effects on the wild-type allele. other disorders our previous (López-Márquez et al., 2022). This point is open for future experiments.

The therapeutic benefit of our approach to correct PKD was assessed in CD34^+^ cells derived from a PKD patient, who carried c.359 C >T and c.1168 G >A mutations. This cell source was selected as a relevant cell type to be used in HSPC gene therapy since genetically corrected HSPCs would engraft in myeloablative conditioned patients and repopulate patient’s hematopoiesis. The HSPC gene therapy for PKD had been shown feasible previously by our group (Garcia-Gomez et al., 2016; Fañanas-Baquero et al., 2021; Navarro et al., 2021) and in the current clinical trial for PKD (NCT04105166) (López Lorenzo et al., 2020). These different gene therapy approaches represent general treatments for PKD patients. However, the combination of gRNA and ssODN is a patient-specific gene therapy approach that should be explored to assess its real therapeutic potential. Interestingly, the ATP content of erythroid cells derived from gene-edited PKD HSPCs showed a slight improvement when were corrected only with one of the mutation-specific gene editing tools or almost a total compensation when both mutations were addressed simultaneously (Figure 4B). Surprisingly, we could only detect a small frequency of precise correction in c. 1168 mutation (Figure 4C). This discrepancy might be attributed to the fact that the terminal erythroid cells lack the nucleus and consequently, the genomic analysis was done in the remaining not fully erythroid differentiated cells, which might be the uncorrected ones and the most affected by RPK defects. This point should be confirmed when more PKD CD34^+^ cells are accessible, and the genomic analysis should be done at different time points over the *in vitro* erythroid differentiation. Additionally, RPK works as a tetramer, so the unfunctional monomers produced from the more severe mutated alleles might be eliminated by the high frequency of InDels around the mutations and/or favored its displacement from the tetramer by less frequent but more stable gene edited RPK monomers. This is consistent with the fact that Asp^390^ is important for the allosteric regulation of RPK, suggesting that the c.1168 G >A mutation may lock the protein in an inactive conformation (Valentini et al., 2002), and therefore that the elimination of this mutated form might restore RPK tetramer activity, since the tetramers will be formed by monomers with the less severe alteration and corrected one.

Overall, our results demonstrate the feasibility of a highly personalized gene editing therapy, combining mutation-specific gRNAs with ssODNs to treat PKD-causing mutations. Increase in the efficacy and mutation specificity must be carried out to achieve the clinical relevance required for this promising approach to correct PKD.

## Data Availability

The original contributions presented in the study are included in the article/Supplementary Material, further inquiries can be directed to the corresponding authors.
