# Supplementary material for: Specific correction of pyruvate kinase deficiency-causing point mutations by CRISPR/Cas9 and single-stranded oligodeoxynucleotides
Source: Front Genome Ed. 2023 Apr 28;5:1104666. doi: 10.3389/fgeed.2023.1104666 (PMC10175809; doi:10.3389/fgeed.2023.1104666)
Supplement: Supplementary file 1 [file DataSheet1.pdf]

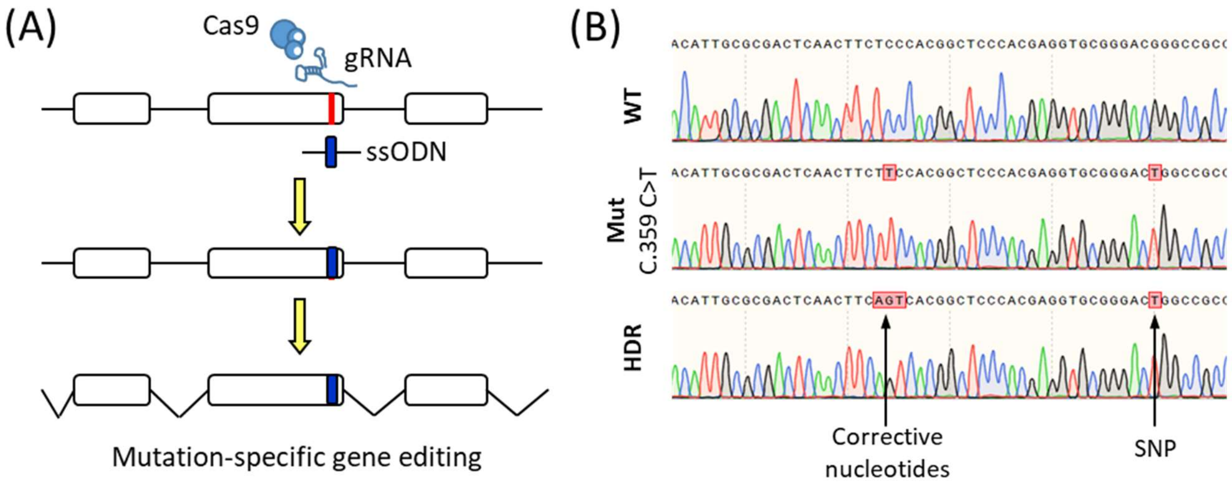

SUPPLEMENTARY FIGURE 1. Correction of the c.359 C>T point mutations present in PKD-derived lymphoblastic cell line. **(A)** Diagram shows the gene editing strategy to precisely correct PKD mutations. **(B)** Sanger Sequencing from Zero-Blunt cloned wild-type allele (top panel), c.359 C>T mutated allele (middle panel), and the precise gene edited allele (HDR, bottom panel).

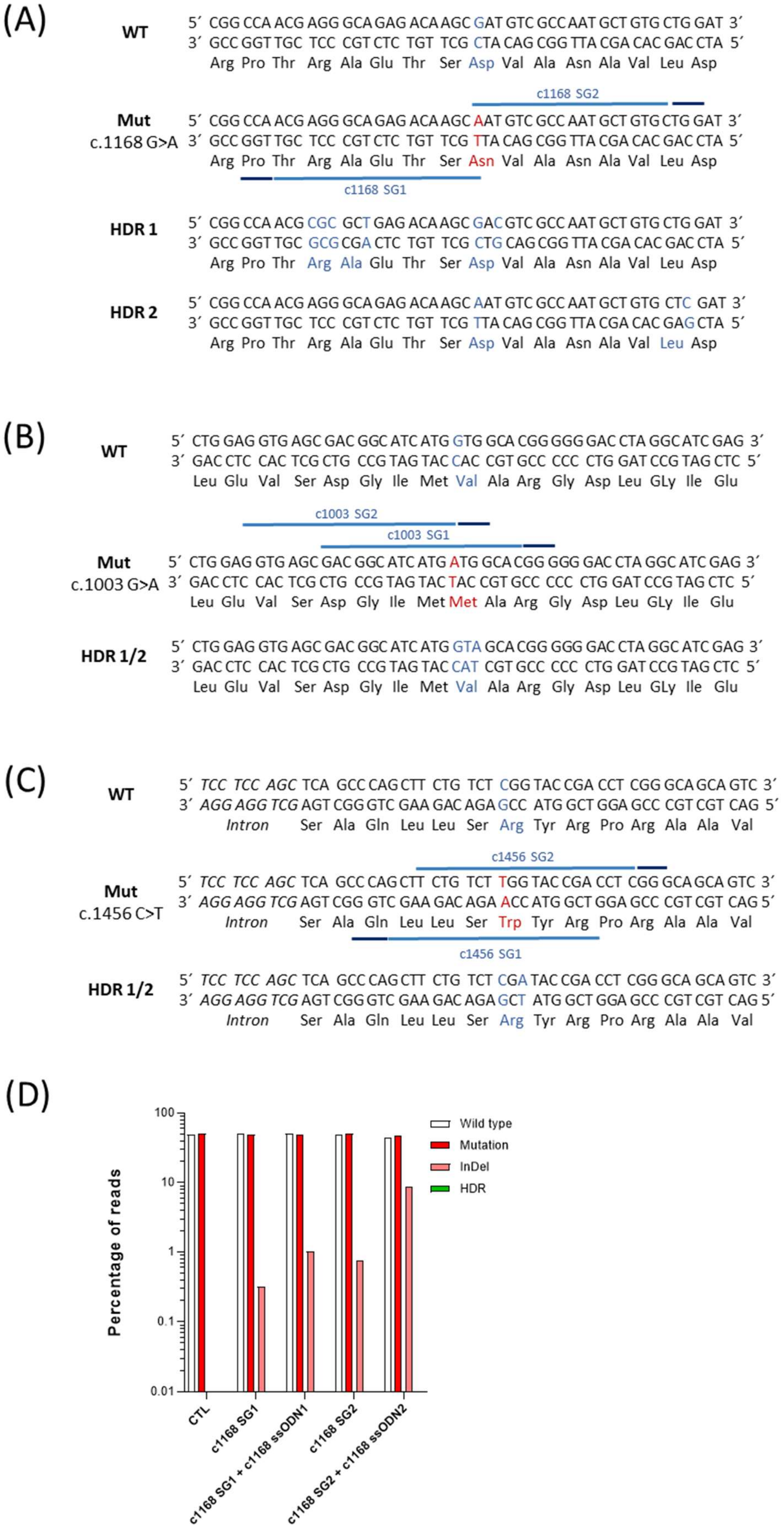
(A)
WT
5´ CGG CCA ACG AGG GCA GAG ACA AGC GAT GTC GCC AAT GCT GTG CTG GAT 3´
3´ GCC GGT TGC TCC CGT CTC TGT TCG CTA CAG CGG TTA CGA CAC GAC CTA 5´
Arg Pro Thr Arg Ala Glu Thr Ser Asp Val Ala Asn Ala Val Leu Asp
c1168 SG2
Mut
c.1168 G>A
5´ CGG CCA ACG AGG GCA GAG ACA AGC AAT GTC GCC AAT GCT GTG CTG GAT 3´
3´ GCC GGT TGC TCC CGT CTC TGT TCG TTA CAG CGG TTA CGA CAC GAC CTA 5´
Arg Pro Thr Arg Ala Glu Thr Ser Asn Val Ala Asn Ala Val Leu Asp
c1168 SG1
HDR 1
5´ CGG CCA ACG CGC GCT GAG ACA AGC GAC GTC GCC AAT GCT GTG CTG GAT 3´
3´ GCC GGT TGC GCG CGA CTC TGT TCG CTG CAG CGG TTA CGA CAC GAC CTA 5´
Arg Pro Thr Arg Ala Glu Thr Ser Asp Val Ala Asn Ala Val Leu Asp
HDR 2
5´ CGG CCA ACG AGG GCA GAG ACA AGC AAT GTC GCC AAT GCT GTG CTC GAT 3´
3´ GCC GGT TGC TCC CGT CTC TGT TCG TTA CAG CGG TTA CGA CAC GAG CTA 5´
Arg Pro Thr Arg Ala Glu Thr Ser Asp Val Ala Asn Ala Val Leu Asp
(B)
WT
5´ CTG GAG GTG AGC GAC GGC ATC ATG GTG GCA CGG GGG GAC CTA GGC ATC GAG 3´
3´ GAC CTC CAC TCG CTG CCG TAG TAC CAC CGT GCC CCC CTG GAT CCG TAG CTC 5´
Leu Glu Val Ser Asp Gly Ile Met Val Ala Arg Gly Asp Leu GLy Ile Glu
c1003 SG2
c1003 SG1
Mut
c.1003 G>A
5´ CTG GAG GTG AGC GAC GGC ATC ATG ATG GCA CGG GGG GAC CTA GGC ATC GAG 3´
3´ GAC CTC CAC TCG CTG CCG TAG TAC TAC CGT GCC CCC CTG GAT CCG TAG CTC 5´
Leu Glu Val Ser Asp Gly Ile Met Met Ala Arg Gly Asp Leu GLy Ile Glu
HDR 1/2
5´ CTG GAG GTG AGC GAC GGC ATC ATG GTA GCA CGG GGG GAC CTA GGC ATC GAG 3´
3´ GAC CTC CAC TCG CTG CCG TAG TAC CAT CGT GCC CCC CTG GAT CCG TAG CTC 5´
Leu Glu Val Ser Asp Gly Ile Met Val Ala Arg Gly Asp Leu GLy Ile Glu
(C)
WT
5´ TCC TCC AGC TCA GCC CAG CTT CTG TCT CGG TAC CGA CCT CGG GCA GCA GTC 3´
3´ AGG AGG TCG AGT CGG GTC GAA GAC AGA GCC ATG GCT GGA GCC CGT CGT CAG 5´
Intron Ser Ala Gln Leu Leu Ser Arg Tyr Arg Pro Arg Ala Ala Val
c1456 SG2
Mut
c.1456 C>T
5´ TCC TCC AGC TCA GCC CAG CTT CTG TCT TGG TAC CGA CCT CGG GCA GCA GTC 3´
3´ AGG AGG TCG AGT CGG GTC GAA GAC AGA ACC ATG GCT GGA GCC CGT CGT CAG 5´
Intron Ser Ala Gln Leu Leu Ser Trp Tyr Arg Pro Arg Ala Ala Val
c1456 SG1
HDR 1/2
5´ TCC TCC AGC TCA GCC CAG CTT CTG TCT CGA TAC CGA CCT CGG GCA GCA GTC 3´
3´ AGG AGG TCG AGT CGG GTC GAA GAC AGA GCT ATG GCT GGA GCC CGT CGT CAG 5´
Intron Ser Ala Gln Leu Leu Ser Arg Tyr Arg Pro Arg Ala Ala Val
(D)
Percentage of reads
100
10
1
0.1
0.01
Wild type
Mutation
InDel
HDR
CTL
c1168 SG1
c1168 SG1 + c1168 ssODN1
c1168 SG2
c1168 SG2 + c1168 ssODN2

7

SUPPLEMENTARY FIGURE 2. Correction of different point mutations present in PKD patients. **(A)** Genomic sequences of the wild-type allele show the wild-type nucleotide and amino acid in blue (top panel), mutated allele with the c.1168 G>A mutation and the mutated amino acid in red (middle panel) with the target sequences of the two gRNA targeting the mutation (light blue line) and their PAM sequences (dark blue line), and the precise gene editing output with the modified nucleotides and reverted amino acid in blue (bottom panel). **(B)** Genomic sequences of the wild-type allele show the wild-type nucleotide and amino acid in blue (top panel), mutated allele with the c.1003 G>A mutation and the mutated amino acid in red (middle panel) with the target sequences of the two gRNA targeting the mutation (light blue line) and their PAM sequences (dark blue line), and the precise gene editing output with the modified nucleotides and reverted amino acid in blue (bottom panel). **(C)** Genomic sequences of the wild-type allele show the wild-type nucleotide and amino acid in blue (top panel), mutated allele with the c.1456 C>T mutation and the mutated amino acid in red (middle panel) with the target sequences of the two gRNA targeting the mutation (light blue line) and their PAM sequences (dark blue line), and the precise gene editing output with the modified nucleotides and reverted amino acid in blue (bottom panel). **(D)** Frequency of the different reads identified by Next Generation Sequencing in PKD-LCLs gene-edited with the specific gene editing tools for c.1168 G>A mutation.

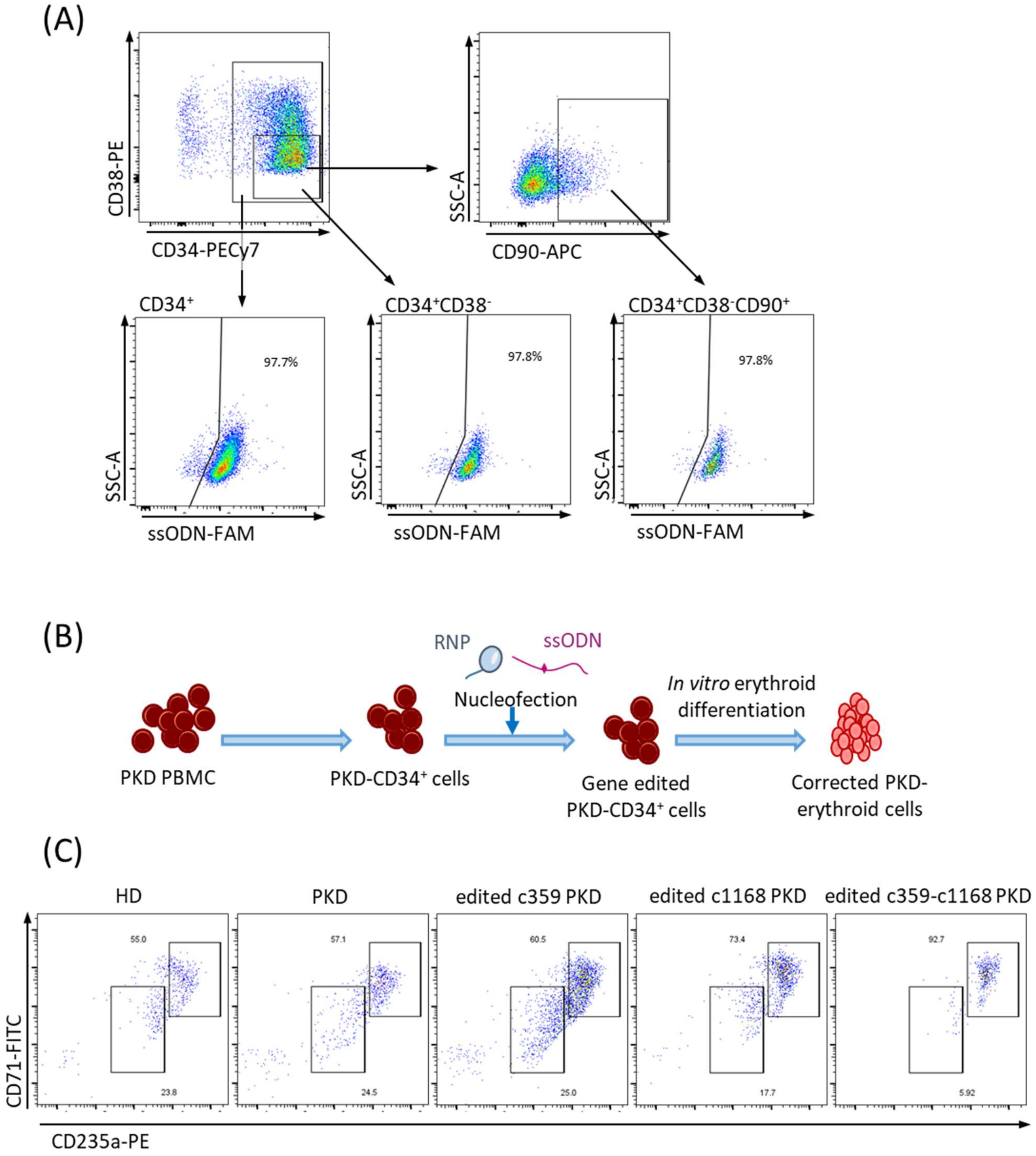

SUPPLEMENTARY FIGURE 3. Gene editing in hematopoietic stem and progenitor cells (HSPCs) with gRNA and ssODN targeting PKD-causing mutations. **(A)** Flow cytometry analysis of HSPCs nucleofected with ssODN-FAM in the $CD34^{+}$, $CD34^{+}CD38^{-}$ and $CD34^{+}CD38^{-}CD90^{+}$ subpopulations. **(B)** The scheme shows the gene editing and erythroid differentiation of PKD-HSPCs to correct c.359 C>T and c.1168 G>A mutations. **(C)** Flow cytometry analysis of the erythroid cells in vitro

differentiated from PKD-HSPCs after gene editing with the specific tools for c.359 C>T and c.1168 G>A mutations.

Supplementary Table S1. List of gRNAs and ssODNs

Mutation	SG name	SG sequence	ssODN name	ssODN sequence
NM_00298.6: C.359 C>T	c359 SG1	CGCGACTCAACTTCTTCCA	c359 ssODN	ATGATCAAGGCCGGGATGAACATTGCGCGACTCAACTTCAGTCACGGCTCCCACGA GGTGCGGGACGGGCCGCCGGGCAGT
	c359 SG2	TTCTTCCACGGCTCCCACG		
NM_00298.6: C.1168 G>A	c1168 SG1	TGCTTGTCTCTGCCCTCGT	c1168 ssODN 1	CCCAGATGCTGGAGAGCATGATTACCAAGCCCCGGCCAACGCGCGCTGAGACAAG CGACGTCGCCAATGCTGTGCTGGATG
	c1168 SG2	AATGTCGCCAATGCTGTGC	c1168 ssODN 2	GGCCAACGAGGGCAGAGACAAGCAATGTCGCCAATGCTGTGCTCGATGGGGCTGA CTGCATCATGCTGTCAGGGGAGACTG
NM_00298.6: c.1003 G>A	c1003 SG1	GACGGCATCATGATGGCACG	c1003 ssODN 1+	CAGGTTTGATGAAATCCTGGAGGTGAGCGACGGCATCATGGTAGCACGGGGGGAC CTAGGCATCGAGATCCCAGCAGAGAAGGTT
			c1003 ssODN 1-	AACCTTCTCTGCTGGGATCTCGATGCCTAGGTCCCCCCGTGCTACCATGATGCCGTC GCTCACCTCCAGGATTTCATCAAACCTG
	c1003 SG2	GGTGAGCGACGGCATCATGA	c1003 ssODN 2+	CTCAGGTTTGATGAAATCCTGGAGGTGAGCGACGGCATCATGGTAGCACGGGGGG ACCTAGGCATCGAGATCCCAGCAGAGAAGG
			c1003 ssODN 2-	CCTTCTCTGCTGGGATCTCGATGCCTAGGTCCCCCCGTGCTACCATGATGCCGTCGC TCACCTCCAGGATTTCATCAAACCTGAG
NM_00298.6: c.1456 C>T	c1456 SG1	GTCGGTACCAAGACAGAAGC	c1456 ssODN 1+	CTGGGCTGACCTTCTCTGCCTCCTCCAGCTCAGCCCAGCTTCTGTCTCGATACCGACC TCGGGCAGCAGTCATTGCTGTCACCCGCTCTG
			c1456 ssODN 1-	CAGAGCGGGTGACAGCAATGACTGCTGCCCGAGGTCGGTATCGAGACAGAAGCTG GGCTGAGCTGGAGGAGGCAGAGAAGGTCAGCCCAG
	c1456 SG2	TCTGTCTTGGTACCGACCTC	c1456 ssODN 2+	GACCTTCTCTGCCTCCTCCAGCTCAGCCCAGCTTCTGTCTCGATACCGACCTCGGGCA GCAGTCATTGCTGTCACCCGCTCTGCCCAGGC
			c1456 ssODN 2-	GCCTGGGCAGAGCGGGTGACAGCAATGACTGCTGCCCGAGGTCGGTATCGAGACA GAAGCTGGGCTGAGCTGGAGGAGGCAGAGAAGGTC

Supplementary Table S2. List of Next-Generation Sequencing primers

Mutation	Primer name	Primer sequence
NM_00298.6: C.359 C>T	c359 PKLR F1 NGS	ACACTCTTTCCCTACACGACGCTCTTCCGATCTCTGGGGGAACGTTGTCTGAA
	c359 PKLR R1 NGS	GACTGGAGTTCAGACGTGTGCTCTTCCGATCTATCCTCCTGCCCCACCCACT
NM_00298.6: C.1168 G>A	c1168 PKLR F1 NGS	ACACTCTTTCCCTACACGACGCTCTTCCGATCTATTTTGGGACACTCTGAGAGTGT
	c1168 PKLR R1 NGS	GACTGGAGTTCAGACGTGTGCTCTTCCGATCTTGACAGCATGATGCAGTCAG
NM_00298.6: c.1003 G>A	c1003 PKLR F2 NGS	ACACTCTTTCCCTACACGACGCTCTTCCGATCTCTTCGGCCCTGTCGCTATT
	c1003 PKLR R2 NGS	GACTGGAGTTCAGACGTGTGCTCTTCCGATCTGCAGCGCCCAATCATCATCT
NM_00298.6: c.1456 C>T	c1456 PKLR F3 NGS	ACACTCTTTCCCTACACGACGCTCTTCCGATCTTGTTCTGGGCTGACCTTCTC
	c1456 PKLR R3 NGS	GACTGGAGTTCAGACGTGTGCTCTTCCGATCTGGAGGTTCACGGTAAAGCAA
